# Clinical and Laboratory Predictors of Long-Term Outcomes after Catheter Ablation for a Ventricular Electrical Storm

**DOI:** 10.1155/2024/5524668

**Published:** 2024-02-05

**Authors:** Grzegorz Sławiński, Maja Hawryszko, Julia Dyda-Kristowska, Tomasz Królak, Maciej Kempa, Dariusz Świetlik, Dariusz Kozłowski, Ludmiła Daniłowicz-Szymanowicz, Ewa Lewicka

**Affiliations:** ^1^Department of Cardiology and Electrotherapy, Medical University of Gdańsk, Smoluchowskiego 17 Street, 80-214 Gdańsk, Poland; ^2^Division of Biostatistics and Neural Networks, Medical University of Gdańsk, Dębinki 1 Street, 80-211 Gdansk, Poland

## Abstract

**Background:**

Ventricular electrical storm (VES) is characterized by the occurrence of multiple episodes of sustained ventricular arrhythmias (VA) over a short period of time. Radiofrequency ablation (RFA) has been reported as an effective treatment in patients with ventricular tachycardia (VT).

**Objective:**

The aim of the present study was to indicate the short-term and long-term predictors of recurrent VA after RFA was performed due to VES.

**Methods:**

A retrospective, single-centre study included patients, who had undergone RFA due to VT between 2012 and 2021. In terms of the short-term (at the end of RFA) effectiveness of RFA, the following scenarios were distinguished: complete success: inability to induce any VT; partial success: absence of clinical VT; failure: inducible clinical VT. In terms of the long-term (12 months) effectiveness of RFA, the following scenarios were distinguished: effective ablation: no recurrence of any VT; partially successful ablation: VT recurrence; ineffective ablation: VES recurrence.

**Results:**

The study included 62 patients. Complete short-term RFA success was obtained in 77.4% of patients. The estimated cumulative VT-free survival and VES-free survival were, respectively, 28% and 33% at the 12-month follow-up. Ischemic cardiomyopathy and complete short-term RFA success were predictors of long-term RFA efficacy. Neutrophil to lymphocyte ratio (NLR) and GFR <60 mL/min/1.73 m^2^ were associated with VES recurrence. NLR ≥2.95 predicted VT and/or VES recurrence with a sensitivity of 66.7% and specificity of 72.2%.

**Conclusion:**

Ischemic cardiomyopathy and short-term complete success of RFA were predictors of no VES recurrence during the 12-month follow-up, while NLR and GFR <60 ml/min/1.73 m^2^ were associated with VES relapse.

## 1. Introduction

Ventricular electrical storm (VES) is a clinical situation characterized by three or more episodes of sustained ventricular tachycardia (sVT) occurring within 24 hours [1]. The triad of a susceptible electrophysiological substrate, triggers, and autonomic dysregulation take part in the pathogenesis of VES [2]. Treatment includes correction of the underlying abnormalities (electrolyte disturbances, coronary angioplasty of the artery responsible for myocardial ischemia), antiarrhythmic drugs, sedative medications, and in some patients also catheter ablation. Radiofrequency ablation (RFA) has been reported as an effective treatment in patients with ventricular tachycardia (VT). Therapy of ventricular tachycardia differs depending on whether we are dealing with monomorphic ventricular tachycardia (an important role of transcatheter ablation) or with polymorphic ventricular tachycardia (the dominant role of coronary revascularization, correction of electrolyte disturbances, and correction of current pharmacotherapy, avoiding drugs that prolong QTc interval; programming a correspondingly high basic rate in the case of patients with an implanted pacemaker/implantable cardioverter-defibrillator). It should be emphasized, however, that also in the case of polymorphic arrhythmias, with confirmation of the trigger (repetitive, monomorphic ventricular premature complexes), it is possible to perform effective transcatheter ablation. When present, substrate ablation targeting scar is also a reasonable option even if premature ventricular complexes are absent [3, 4]. There are several studies on the prognostic factors for recurrent VT after the RFA procedure. However, there is little research on the laboratory parameters in predicting recurrent ventricular arrhythmias (VA). The aim of the present study was to indicate the short-term and long-term predictors of recurrent VA after RFA was performed due to VES, with particular emphasis on the laboratory biomarkers.

## 2. Methods

### 2.1. Study Design and Population

Our retrospective study included all patients, who had undergone radiofrequency ablation (RFA) due to VT between 2012 and 2021 at the Department of Cardiology and Electrotherapy Medical University of Gdańsk in Poland. Then, among them, we selected patients, who underwent RFA due to VES.

In patients with implantable cardioverter-defibrillator (ICD), the ventricular electrical storm was recognized when three or more episodes of sustained VT (sVT) occurred in 24 hours, requiring therapy, i.e., antitachycardia pacing (ATP) or shocks, and with at least 5 minutes between these episodes. In patients without implanted ICDs, VES is recognized in the case of at least three separate episodes of sVT in 24 hours [2, 5–8].

The analysis included patients' demographic data, underlying heart disease, concomitant diseases, pharmacological treatment, and selected laboratory parameters obtained from peripheral blood samples at the time of RFA. The neutrophil to lymphocyte ratio (NLR) was calculated by dividing the number of neutrophils by the number of lymphocytes. Chronic renal disease was diagnosed when the glomerular filtration rate (GFR) was <60 mL/min/1.73 m^2^ in two examinations performed within the last 3 months. The CKD-EPI creatinine equation was used to estimate GFR.

### 2.2. Radiofrequency Ablation Procedure

Prior to RFA, potential reversible causes of VES were excluded, including coronary angiography in patients with ischemic cardiomyopathy and elevated troponin levels. Endocardial access was obtained to the right ventricle using a transvenous approach and to the left ventricle using a transseptal or retrograde transaortic approach. In all procedures involving left-sided access, the systemic anticoagulation was achieved with heparin with an initial bolus of 5000 U administered intravenously, followed by infusion of 1000–2000 U/hour or based on ACT >300 s). Endocardial electrogram and surface ECG were recorded using the CardioLab EP recording system (General Electric, Houston, TX, USA). Electrical stimulation was delivered using an external stimulator (Qubic Stim Cardiac Stimulator, Biotronik, Berlin, Germany).

In the case of hemodynamically stable VT or in the absence of availability of 12-lead ECG (only IEGM recordings), conventional mapping strategies such as activation and entrainment mapping were used. The goal was to identify the critical isthmus. Substrate-based strategies were applicable to hemodynamically unstable VT and included ablation of late potentials and scar homogenization/local abnormal ventricular activity (LAVA) elimination.

Programmed ventricular stimulation was used to induce VT, with a basal pacing cycle length of 600 ms and 400 ms, and with up to three extrastimuli. Voltage mapping was performed during sinus or paced rhythm from an inserted catheter, using a three-dimensional electro-anatomic navigation system CARTO 3 (Biosense Webster, Diamond Bar, CA). Endocardial areas with bipolar electrogram amplitudes of <0.5 mV were defined as zones of dense scar tissue, areas with amplitudes of 0.5 to 1.5 mV corresponded with scar border zone, and areas >1.5 mV delineated normal endocardial tissue [9]. Sites with fractionated electrograms or late potentials within or adjacent to areas of the scar were also identified and tagged. Pacemapping was performed in areas with abnormal electrograms and in the sites in which the VT exit (reentry mechanism) or VT origin (focal mechanism) was suspected on the base of the 12-lead ECGs of VT. The target for ablation was defined as all areas potentially involved in the arrhythmia mechanism based on the mapping technique described above [10–13].

Radiofrequency current was delivered with a 3.5-mm open irrigated catheter using power settings of 30 to 40 W up to 60 seconds or an ablation index of 500–600 (ablations performed in 2020-2021). The ablation index (AI) is an index that incorporates contact force, time, and radiofrequency power simultaneously and is able to predict lesion size and outcomes in RFA. The ablation index was used in some patients as an additional marker of application time, especially in spots when adequate contact force (>10 g) was not achieved. In those applications, RF delivery was prolonged above 60 seconds if the ablation index was <500–600. According to our knowledge, there is only one ongoing clinical trial to assess the value of AI in VT ablation (ClinicalTrials.gov Identifier: NCT03437408). The value of 500–600 used in our study was established arbitrarily based on the assumption that it should be higher than used in pulmonary vein isolation (400–550) and based on our observations that AI = 500–600 is the value reached during 60 seconds application of 30−40 W in spots with good contact (>10 g).

There are reports in the literature that the ablation index value during ablation may be a predictor of the effectiveness of ablation of ventricular premature complexes. The maximum and mean AI values were statistically higher in the RFA success group (median of the maximum AI 630 (IQR 561–742); median of the mean AI 489 (IQR 411–560)), in which authors assessed RFA efficacy in premature ventricular complex ablation [14]. It should be emphasized that AI was only an additional parameter monitored during the RFA. It has a limited value for guiding VT ablation, which may be related to small proportional significance of application duration and complex tissue architecture [15].

After ablation, right ventricular programmed stimulation was performed to assess the effect of catheter ablation. In some patients, the bipolar remap of the ablation area was made to assess late/fragmented potentials abolition or pacing from inside the lesion was performed to check failure to capture the ventricle.

### 2.3. Study Endpoints

The endpoints assessed in the study were divided into two types: in terms of short-term effectiveness of RFA and in terms of long-term effectiveness of RFA.

Short-term efficacy was assessed immediately after the completion of ablation and consisted in assessing the possibility of induction of VT with the protocol as before ablation. There were three scenarios available at that time.

Complete short-term RFA success was defined as the inability to induce any VT (clinical or other) at the end of the RFA procedure, while partial success was defined as the absence of clinical VT. RFA failure was recognized when the clinical VT has been induced after the RFA procedure.

Long-term efficacy was assessed 12 months after RFA based on an outpatient clinic visit. Routine follow-up of patients after RFA in our centre includes a visit to the hospital outpatient clinic and control of the cardiac implantable electronic device (CIED) at 3, 6, and 12 months after RFA. All VA episodes recorded by the CIED were analyzed in this study, and those lasting >30 sec or terminated by the CIED were taken into account.

In terms of the long-term effectiveness of RFA, the following scenarios were distinguished:

1. Effective ablation: no recurrence of any VT within 12 months after RFA
2. Partially successful ablation: VT recurrence within 12 months after RFA
3. Ineffective ablation: VES recurrence within 12 months after RFA

### 2.4. Statistical Analysis

Continuous variables were expressed as the mean ± SD if normally distributed or median if not normally distributed. In the case of continuous variables, normal distribution was tested by using the 1-sample Kolmogorov–Smirnov test. Categorical data were expressed as numbers and percentages. Continuous variables were compared using independent-sample parametric (unpaired Student *t*) or nonparametric (Mann–Whitney *U*, Kruskal–Wallis test by ranks) tests. Categorical variables were compared using the chi-square test or the Fisher exact test when appropriate. Survival curves were generated using the Kaplan–Meier method and compared using the log-rank test. Univariate and multivariate Cox proportional hazards analyses were used to test the association between the outcome events and baseline covariates. For multivariate analysis, only variables with a *p* value ≤0.15 in the univariate analysis were included. Stepwise regression was used in multivariate analysis to determine independent risk factors of VES recurrence. The area under the receiver operating characteristic curve (AUC) was used to determine the cut-off value of the independent variable which predicted the recurrence of VT and/or VES after RFA. Two-tailed tests were considered statistically significant at the 0.05 level. Data were analyzed with the use of STATISTICA 13 software, licensed for the Medical University of Gdansk, Poland.

The study was retrospective in nature, it did not require the participation of patients, and therefore, the consent of the bioethics committee was not required for its conduct.

## 3. Results

The study included 56 men (90.3%) and 6 women (9.7%) with a mean age of 65.7 years at the time of RFA. The baseline characteristics of the study population are shown in Table 1. Ischemic cardiomyopathy was recognized in 73% (*n* = 45), while dilated cardiomyopathy in 19.4% (*n* = 12) of patients. Arrhythmogenic right ventricular cardiomyopathy (ARVC) and left ventricular noncompaction (LVNC) were less common (3 and 2 patients, respectively). The majority of patients were diagnosed with heart failure with reduced ejection fraction (HFrEF): 54 (87.1%), while the mean left ventricular EF in the study group was 28.4% ± 9.7%. 32 (51.6%) patients were taking amiodarone at the time of RFA. Amiodarone was continued in all patients who had received this drug prior to RFA. During the follow-up period, there were no complications related to the use of this type of treatment. 60 patients (97%) had an ICD implanted.

The mean rate of clinical VT was 151 ± 29 bpm. The mean RFA procedure duration was 218 ± 60 min. During the electrophysiological study (EPS), the median number of different types of VT induced was 2. Two patients underwent both, the endo- and epicardial RFA. Among the patients requiring LV RFA, retroaortic access was used in 44% of patients, while transseptal access was used in 56%. Two patients underwent RFA in general anesthesia, and these were patients with long-term ineffective RFA, moreover, patients who died during the follow-up.

The majority (69.4%) of induced clinical VTs were hemodynamically stable. All VTs qualified for RFA were monomorphic arrhythmias. Serious complications of RFA occurred in 6 patients (9.7%). It was a permanent (2 cases) or transient third-degree atrioventricular block (1 case), ischemic stroke (1 case), pulmonary oedema requiring respiratory therapy ended in death in the mechanism of pulseless electrical activity (1 case), and aorto-atrial fistula (1 case). Aorto-atrial fistula was a complication found in a 69-year-old patient with ischemic cardiomyopathy, dextrocardia, and visceral inversion (Supplementary Figure 1). During the ablation, the right atrial wall was punctured into the aortic root. Transesophageal echocardiography did not reveal any fluid in the pericardium, but a slight leak from the aorta into the right atrium was observed (three mm diameter), which was confirmed in the subsequent examination two days later. After a cardiac surgery consultation, it was decided to implement conservative treatment. Nine days after ablation, a computed tomography of the aorta was performed, which confirmed the closure of the fistula. The clinical characteristics of patients with serious RFA complications are presented in Table 2.

### 3.1. Short-Term Outcomes

Complete RFA success was obtained in 48 (77.4%) patients, while in 12 (19.4%) patients, it was a partial success. Only in 2 (3.2%) patients, RFA failure was noted. Among the assessed laboratory parameters, only a significantly higher concentration of ALT was found in patients with RFA failure (*p*=0.005), as shown in Table 3. Due to the small size of the subgroup with an RFA failure, the logistic regression analysis was not performed in determining predictors of RFA failure.

### 3.2. Long-Term Outcomes

**Figure 1:**
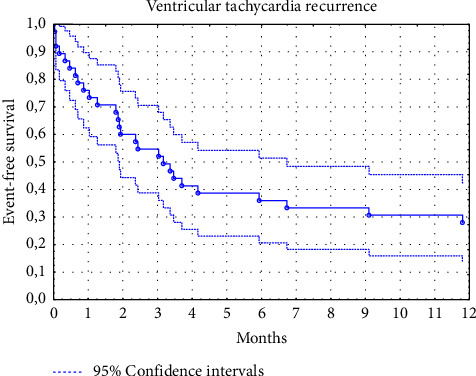
Patients free from ventricular tachycardia (VT) recurrence (Kaplan–Meier curve) during the 12-month follow-up after the radiofrequency ablation performed due to ventricular electrical storm (*N* = 47).

**Figure 2:**
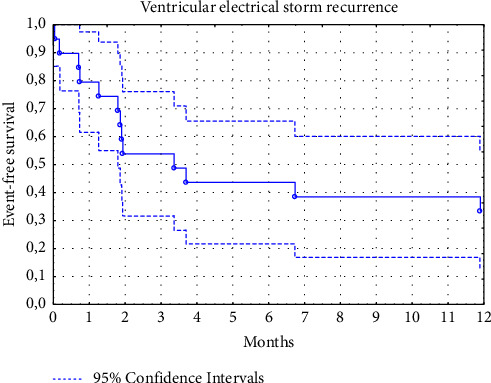
Kaplan–Meier curve representing patients free from ventricular electrical storm (VES) recurrence during the 12-month follow-up after the radiofrequency ablation performed due to VES (*N* = 47).

**Figure 3:**
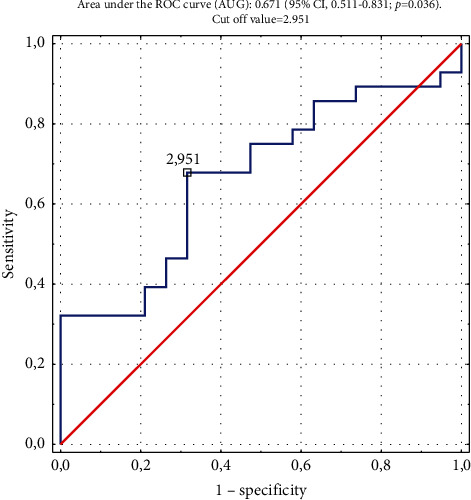
Threshold of the preablation neutrophil to lymphocyte ratio (NLR) that determined an increased risk of ventricular tachycardia (VT) and/or ventricular electrical storm (VES) recurrence after the radiofrequency ablation performed for VES.

Median follow-up was 14 months (range, 1 to 100 months); however, in 15 patients (24%), data on the long-term outcomes of the RFA were not obtained because they did not come for the control visits to our centre, and contact with them was lost. Therefore, the final analysis of the long-term results of RFA included data from 47 patients. In the remaining patients, there were no recurrences of VT or VES in 19 (40.4%) patients (effective ablation). In 15 (31.9%) patients, VT recurrences were recorded but without electrical storm (partially successful ablation), and only 13 patients (27.7%) had a recurrence of VES (ineffective ablation). The median time to recurrence of VT was 3.2 months, and it was 3.4 months in the case of VES relapse. The estimated cumulative VT-free survival was 76% (95% CI, 62 to 90), 54% (95% CI, 38 to 70), and 31% (95% CI, 16 to 45) at the 1-, 3-, and 12-month follow-up, respectively (Figure 1). The estimated cumulative VES-free survival was 79% (95% CI, 62 to 97), 54% (95% CI, 32 to 76), and 38% (95% CI, 16 to 60%) at the 1-, 3-, and 12-month follow-up, respectively (Figure 2). Among patients in the partially successful ablation group, the ICD therapies identified in patients with recurrent VT were ATP/shocks/ATP + shocks in, respectively, 6/6/3 patients, and in the ineffective ablation group, ATP/shocks/ATP + shocks in, respectively, 4/3/6 patients. No inadequate ICD interventions were noted during follow-up. Comparison of laboratory parameters before RFA for VES depending on the long-term ablation result is presented in Table 4. Results of the univariate and multivariate Cox proportional hazards analyses to determine independent predictors of VES recurrence after RFA are presented in Table 5. In multivariate analysis ischemic cardiomyopathy, the underlying heart disease and complete short-term RFA success were independent predictors of the long-term RFA efficacy. In contrast, neutrophil to lymphocyte ratio and GFR <60 mL/min/1.73 m^2^ were independently associated with VES recurrence during the 12-month follow-up (central illustration). NLR ≥2.95 assessed before ablation (Figure 3) predicted VT and/or VES recurrence with a sensitivity of 66.7% and a specificity of 72.2% (the area under the ROC curve for NLR (AUC) in predicting VT/VES recurrence was 0.671 (95% CI, 0.511–0.831; *p*=0.036).

**Figure 4:**
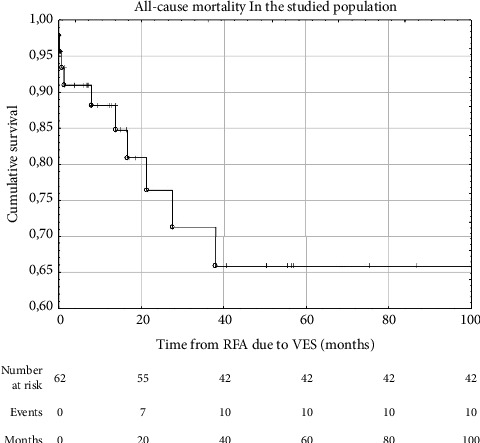
Survival in the studied patients (*N* = 62) after the radiofrequency ablation (RFA) for the ventricular electrical storm (VES) (Kaplan–Meier curve).

During the follow-up, 10 from 62 (16.1%) patients died. Among the causes of death, end-stage, severe heart failure dominated (*n* = 8), and in the remaining cases, it was gastrointestinal obstruction and septic shock.  Figure 4 shows the Kaplan–Meier curve representing all-cause mortality in the studied population. Among the assessed laboratory parameters, only the concentration of hsTnI before ablation correlated significantly with the risk of death (HR 1.31; CI 1.06–1.6; *p*=0.04).

## 4. Discussion

In the present study, we examined short-term and long-term outcomes of RFA performed in patients with VES, with particular emphasis on predictors of ventricular arrhythmia recurrence after RFA. We found that (1) lack of complete short-term RFA success and ischemic cardiomyopathy (vs. others) were independent predictors of VES recurrence during the 12-month follow-up; (2) among laboratory parameters, the independent predictors also increased neutrophil to lymphocyte ratio and GFR < 60 ml/min/1.73 m^2^; (3) there was a significant correlation between the baseline hsTnI concentration and the risk of death in patients undergoing RFA due to VES.

In a retrospective study, the short-term efficacy of RFA due to electric storm was assessed in 62 patients, while the long-term efficacy was assessed in 47 patients (15 patients were lost to follow-up). The size of the population covered by the study is not large; although due to the relatively rare occurrence of this disease and thus the rare performance of RF ablation in ES, there are reports in the literature on similar groups of patients [16–19] and only several publications on larger groups [20, 21].

The clinical characteristics of patients in our group were similar to those reported by other authors. As in the study by Carbucicchio et al. [22], men were more frequently referred for RFA due to VES (90.3% of our patients vs. 86.7% in the Cabucicchio group), the underlying disease was mainly ischemic cardiomyopathy (70.9% vs. 76%, respectively), and patients presented with decreased left ventricular systolic function (mean LVEF 28.4% vs. 36%, respectively) and moderately fast ventricular tachycardia (151 bpm vs. 157 bpm respectively).

The duration of the ablation procedure (218 min vs. 260 min) and the median number of induced VTs (2 vs. 2) were also similar to those reported by other authors [22]. Unfortunately, due to the retrospective nature of the study, we did not have information on more detailed characteristics of RFA procedures. This is important because, in The VISTA Randomized Multicenter Trial, an extensive substrate-based ablation approach is superior to ablation targeting only clinical and stable VTs in patients with ischemic cardiomyopathy (who constituted most of our material) presenting with well-tolerated VT [23].

Ablation performed due to VES is associated with a relatively low number of serious complications, especially considering the usually severe condition of the patients undergoing this procedure. In our group, we reported serious complications in 9.7% of patients treated with RFA, which was higher than in other studies: 3–6.2% [24–26]. However, unlike other authors, we considered a transient complete atrioventricular block as a serious (not minor) complication. We have not recorded any deaths related to the RFA procedure. Fortunately, this fatal complication is very rare, as documented in other studies [27, 28]. Regarding the short-term results of the RFA, it has been reported in patients with ICM that complete RFA success was achieved in 60–80% of patients, partial success in 15–25%, and failure was reported in 0–10% of patients [29]. As the majority of our study group participants were patients with ICM, we obtained similar results (77.4%, 19.4%, and 9.68%, respectively).

In the long-term assessment of the RFA effect, most of the authors reported that the complete success of the procedure was 62%–100% [25, 30–34]. However, in the study by Kozeluhova et al., it was 48% [35]. In our whole group, RFA was effective in 40% of patients, but unfortunately, we did not have data on this subject in 15 patients who did not come to our centre for postoperative follow-up. On the other hand, the percentage of patients with recurrence of VES was 28%, and in other studies, it was 0–35% [25, 35–37]. It should be emphasized that ablation performed due to VES is a complex procedure, and the relatively high VT or VES recurrence rate reflects the presence of a large myocardial substrate and difficulties in its modification, as suggested by some authors [27].

However, with a low risk of serious complications and often high effectiveness, the RFA procedure is performed in patients with implantable cardioverter-defibrillator (ICD) and recurrent VT. Sapp et al. reported that in patients with ischemic cardiomyopathy and an ICD who had VT despite antiarrhythmic drug therapy, the primary composite outcome of death, VES, or appropriate ICD shock occurred significantly less frequently if they underwent RF ablation, compared to an escalation of antiarrhythmic drug therapy [38]. An interesting option may be stereotactic arrhythmia radioablation (STAR). It is an alternative for patients who, despite optimal pharmacotherapy and the applied RF ablation, still have recurrences of VT/VES [39].

Analyzing the clinical parameters of our patients at the time of RFA, we showed on the basis of a multivariate analysis that ischemic cardiomyopathy as the underlying disease and short-term complete success of RFA were independent predictors of the lack of VES recurrence in the 12-month follow-up. These data confirm the findings from other authors [22] who also reported a higher risk of VES recurrence in patients with nonischemic cardiomyopathy and in those with the unfavorable early effect of RFA. However, it should be underlined that the prognosis of patients with nonischemic cardiomyopathy has improved significantly since the introduction of epicardial ablations, as reported in more recent multicentre studies. On their basis, it is clearly visible that the differences in the frequency of VES relapses disappear among patients with ischemic and nonischemic cardiomyopathy when using both endo- and epicardial ablations [40]. Other reported clinical predictors of recurrent ventricular arrhythmias after RFA due to VES are older age, more severe left ventricular dysfunction, higher NYHA class, amiodarone treatment prior (or according to other authors post) to ablation, poorly tolerated VT, the greater number of different inducible VTs, and a history of atrial fibrillation [27, 41–43].

The analysis of laboratory parameters in our patients at the time of RFA indicated the increased NLR and GFR <60/mL/min/1.73 m^2^ as the independent predictors of VES relapse within the 12-month follow-up after RFA. Laboratory biomarkers have been rarely evaluated in terms of the long-term efficacy of RFA performed in the VES therapy. Kozeluhova et al. reported on renal insufficiency as a predictor of the adverse outcome within the 6-month observation after catheter ablation for VES [35]. Also, Özcan et al. indicated the increased creatinine concentration as the risk factor for the recurrence of VES in patients with ischemic cardiomyopathy [44]. However, these observations have not been confirmed in newer, multicentre studies involving larger numbers of patients. Also, the I-VT score developed and validated by Vergara et al. in its final form did not contain chronic kidney disease as a predictor of the recurrence of an electric storm following RFA [45, 46]. In the abovementioned predictive scale of VT recurrence after RFA, one of the key predictors of VT/VES recurrence turned out to be LVEF. In our case, in the multivariate analysis, this parameter was not statistically significant, although there was a trend in lower LVEF values among patients with long-term partially successful ablation and long-term ineffective ablation, compared to patients with long-term effective ablation (*p*=0.071).

Our study is the first to indicate that NLR can be a predictor of ineffective ablation due to VES. NLR is a hematological parameter for systemic inflammation and stress. There is a lot of evidence in the literature supporting the role of inflammation in the pathogenesis of cardiovascular diseases. The studies conducted so far confirm the relationship between NLR and the severity of atherosclerosis [47]. Higher NLR has been reported as an independent predictor of mortality in patients undergoing angiography or cardiac revascularization and in acute decompensated heart failure [48]. Correlations between NLR and acute coronary syndrome risk prediction models such as SYNTAX, GRACE, and GENSINI have been established. The prognostic value of NLR in valvular disease and in patients undergoing valve replacement procedures has been indicated [49]. Based on a meta-analysis, Angkananard et al. showed the association of NLR with coronary artery disease, acute coronary syndromes, stroke, and other composed cardiovascular events [50]. There are several explanations for the relationship between increased NLR and the risk of cardiovascular events. First of all, neutrophils secrete inflammatory mediators that may lead to the degeneration of the vascular wall [51]. On the other hand, lymphocytes are responsible for the regulation of the inflammatory response, in which regulatory T lymphocytes may inhibit atherosclerosis [52]. NLR was found a predictor of incidence, treatment success, recurrence, and thromboembolic complications in patients with atrial fibrillation [53]. In the prospective study on paroxysmal atrial fibrillation (AF), Trivedi et al. indicated that NLR ≥3.08 in patients undergoing catheter ablation due to AF was a predictor of AF recurrence, with a sensitivity of 72.5% and a specificity of 78.6% [54].

NLR is a simple, widely available, and inexpensive biomarker that provides information on both the inflammatory status and the stress response (high neutrophil count reflects subclinical inflammation, while reduced lymphocyte count reflects physiological stress) [55]. In addition, accumulating neutrophils can contribute to atrial remodeling through the release of proinflammatory mediators [56]. The findings from the studies indicating that preablation NLR levels were significantly associated with the ablation outcomes support observations that inflammation plays a role in AF recurrence. Our results may indicate a similar relationship in patients undergoing RFA due to VES. It seems that the accompanying inflammation is primarily conducive to early relapses of arrhythmias after ablations, while in the midterm and long-term perspective in the case of atrial fibrillation, nonpulmonary vein triggers and pulmonary vein reconnections are of key importance. Therefore, it may be worth revisiting the concept of steroids' use for a short time after the RFA procedure for VES, as was performed in some studies after AF ablations studies [57]. However, this concept is a topic for a separate study.

### 4.1. Study Limitations

A relatively small sample size is the study limitation, as well as the lack of follow-up in 15 patients from our group. It makes some statistical calculations difficult and the conclusions drawn should be treated with caution. Our findings are based on a retrospective analysis of patients undergoing ablation in one centre; however, increased experience in performing RFA, as well as the advances in the technical feasibility of RFA procedure over 9 years, should be taken into account. In order to establish a real statistical link between long-term VES/VT recurrence after RFA, it should be persistent/recurrent inflammation with permanently elevated NLR values, and due to the retrospective nature of the study, these data are not provided by the current study.

Another limitation of the study is the significant heterogeneity of the analyzed group of patients, mainly due to the multiple etiologies of cardiomyopathy. Additionally, patients undergoing RFA for nearly 10 years were included in the analysis, and it was a time of intensive development of electrophysiology (ablation index, epicardial ablations) and cardiology (new drugs: angiotensin receptor-neprilysin inhibitor); all this may also be reflected in the obtained results.

The last limitation of the study is the relatively small amount of data on the RFA methodology in our patient group, due to the retrospective nature of the study and reliance on postablation protocols, which often did not include data such as the amount of ablation, low voltage areas, or size of the ablation area covered.

## 5. Conclusions

RF ablation performed for the ventricular electrical storm is a procedure characterized by a relatively low incidence of serious complications. Ischemic cardiomyopathy and short-term complete success of RFA were independent predictors of no VES recurrence during the 12-month follow-up, while neutrophil to lymphocyte ratio and GFR <60 ml/min/1.73 m^2^ were independently associated with VES relapse. Baseline hsTnI concentration, assessed before the ablation, correlated significantly with the overall mortality among patients undergoing RFA for VES.

## Figures and Tables

**Table 1:** Baseline characteristics of the studied population and in relation to the long-term outcome of the radiofrequency ablation (RFA) performed due to ventricular electrical storm.

	Patients included in the study^*∗*^ (*n* = 62)	Long-term effective ablation (*n* = 19)	Long-term partially successful ablation (*n* = 15)	Long-termineffective ablation (*n* = 13)	*p* value
Age, years	65.7 ± 11.6	60.7 ± 12.9	63.5 ± 13.4	68.8 ± 6.6	0.263

Gender, male	56 (90.3%)	17 (89.5%)	13 (86.7%)	11 (84.6%)	0.920

Underlying heart disease:					
ICM	45 (72.6%)	14 (73.7%)	11 (73.3%)	6 (46.2%)	0.291
DCM	12 (19.4%)	3 (15.8%)	3 (20%)	5 (38.5%)	0.308
ARVC	3 (4.8%)	2 (10.5%)	1 (6.7%)	0	0.488
LVNC	2 (3.2%)	0	0	2 (18.3%)	0.065

LVEF, %	28.4 ± 9.7	31.6 ± 10.8	29.3 ± 10.1	24.5 ± 7.4	0.071

Comorbidities:					
HFpEF	1 (1.6%)	1 (5.3%)	0	0	0.471
HFmrEF	3 (4.8%)	0	0	1 (7.7%)	0.263
HFrEF	54 (87.1%)	15 (79%)	14 (93.3%)	12 (92.3%)	0.381
Hypertension	31 (50%)	9 (47.4%)	8 (53.3%)	5 (38.5%)	0.733
Atrial fibrillation	24 (38.7%)	7 (36.8%)	9 (60%)	3 (23.1%)	0.128
Diabetes	21 (33.9%)	2 (10.5%)	6 (40%)	4 (30.8%)	0.129
Chronic renal failure	24 (38.7%)	4 (21.1%)	6 (40%)	6 (46.2%)	0.220
COPD	7 (11.3%)	1 (5.3%)	4 (26.7%)	2 (15.4%)	0.285

Charlson comorbidity index, median	3	3	4	3	0.172

Pharmacotherapy at the time of RFA:					
Amiodarone	32 (51.6%)	7 (36.8%)	11 (73.3%)	9 (69.2%)	0.065
B-blocker	55 (88.7%)	16 (84.2%)	13 (86.7%)	13 (100%)	0.342
ACEI/ARB/ARNI	49 (79%)	15 (78.9%)	10 (66.7%)	13 (100%)	0.262
MRA	36 (58.1%)	10 (52.6%)	7 (46.7%)	9 (69.2%)	0.474
Sotalol	3 (4.8%)	1 (5.3%)	1 (6.7%)	0	0.663
Class I antiarrhythmic drug	3 (4.8%)	1 (5.3%)	0	1 (7.7%)	1.000

Total procedure duration, min	218 ± 60	193 ± 55	218 ± 46	253 ± 72	0.086

Median number of VTs induced during EPS	2	1	2	3	0.269

RF applications location:					
LV anterior	11 (17.7%)	1 (5.3%)	4 (26.7%)	0	0.045
LV posterior	4 (6.5%)	0	1 (6.7%)	1 (7.7%)	0.495
LV lateral	12 (19.4%)	3 (15.8%)	2 (13.3%)	4 (30.8%)	0.449
LV inferior	11 (17.7%)	2 (10.5%)	3 (20%)	3 (23.1%)	0.607
LV apex	16 (25.8%)	5 (26.3%)	5 (33.3%)	2 (15.4%)	0.552
LVOT	5 (8.1%)	1 (5.3%)	2 (13.3%)	2 (15.4%)	0.607
IVS	7 (11.3%)	1 (5.3%)	3 (20%)	2 (15.4%)	0.418
RVOT	2 (3.2%)	1 (5.3%)	0	1 (7.7%)	0.580
RV apex	1 (1.6%)	1 (5.3%)	0	0	0.471

Clinical VT rate, bpm	151 ± 29	145 ± 23	143 ± 28	166 ± 37	0.224

ACEI: angiotensin converting enzyme inhibitor, ARB: angiotensin receptor antagonist, ARNI: angiotensin receptor-neprilysin inhibitor, ARVC: arrhythmogenic right ventricular cardiomyopathy, COPD: chronic obstructive pulmonary disease, DCM: dilated cardiomyopathy, EPS: electrophysiological study, HFmrEF: heart failure with midrange ejection fraction, HFpEF: heart failure with preserved ejection fraction, HFrEF: heart failure with reduced ejection fraction, ICM: ischemic cardiomyopathy, LVEF: left ventricular ejection fraction, LVNC: left ventricular noncompaction, MRA: mineralocorticoid receptor antagonist, RVOT: right ventricular outflow tract, VT: ventricular tachycardia. ^*∗*^The difference between patients included in the study and the number of patients analyzed for long-term effective ablation results from incomplete follow-up (loss of 15 patients).

**Table 2:** Clinical characteristics of patients with serious complications of the radiofrequency ablation (RFA) performed due to ventricular electrical storm.

Variable	P.J.	N.W.	Z.M.	O.C.	M.J.	P.P.
Sex	Male	Male	Female	Male	Male	Male
Age at RFA, years	77	67	64	66	69	55
Complication	Ischemic stroke	Complete AV block	Pulmonary oedema	Complete AV block	Aorto-atrial fistula	Transient complete AV block
Management	Mechanical thrombectomy	ICD program change (including AV delay optimization under echocardiography)	Infusion of pressor amines, respiratory therapy	ICD program change, then ICD VR upgrade to CRT-D	Observation-spontaneousclosure of the fistula	Observation-spontaneousresolution of the AV block
Clinical VT, bpm	185	115	150	210	130	150
Underlying heart disease	ICM	DCM	ICM	DCM	ICM	ICM
LVEF, %	35	30	20	20	20	25
Charlson comorbidity index, median	5	4	5	1	3	2
Follow-up, days	15	38	1	236	59	37
NLR	5.58	1.29	2.32	1.83	3.96	2.05

AV: atrioventricular, CRT-D: implantable cardiac resynchronization therapy (CRT) defibrillator, DCM: dilated cardiomyopathy, ICD: implantable cardioverter defibrillator, ICM: ischemic cardiomyopathy, LVEF: left ventricular ejection fraction, NLR: neutrophil to lymphocyte ratio, RFA: radiofrequency ablation, VT: ventricular tachycardia.

**Table 3:** Laboratory parameters before radiofrequency ablation (RFA) for ventricular electrical storm depending on the short-term ablation result.

	Study population (*n* = 62)	Complete short-termsuccess (*n* = 48)	Partial short-termsuccess (*n* = 12)	RFA failure (*n* = 2)	*p* value
hsTnI, ng/mL (normal range: <0.0156 for women; <0.0342 for man)	0.053 (0.029–0.150)	0.045 (0.024–0.161)	0.052 (0.031–0.076)	0.185 (0.079–0.290)	0.493
CK-MB, ng/mL (normal range: <3.1)	1.4 (0.9–2.6)	1.4 (0.9–3.4)	1.5 (1.1–1.6)	1.3 (1.1–1.4)	0.906
BNP before RFA, pg/mL (normal range: <159)	261 (145–728)	245 (133–482)	458 (168–1273)	526 (268–784)	0.339
BNP at hospital discharge, pg/mL (normal range: <159)	354 (176–495)	262 (159–509)	393 (369–495)	nd	1.000
Creatinine, mg/dL (normal range: 0.55–1.02)	1.17 (1.0–1.47)	1.18 (1.03–1.46)	1.08 (0.91–1.66)	1.2 (1.15–1.26)	0.894
Potassium, mmol/L (normal range: 3.5–5.1)	4.3 (4.0–4.5)	4.3 (4.0–4.5)	4.5 (4.1–4.8)	4.2 (4.2–4.2)	0.603
CRP, mg/L (normal range: <5.0)	3.4 (1.2–13.3)	3.7 (1.3–12.7)	2.2 (0.9–11.9)	7 (0.6–13.9)	0.782
Neutrophils × 10^9^/L (normal range: 2.0–7.0)	4.9 (3.6–6.6)	5.1 (3.6–7.1)	4.8 (3.5–5.4)	5.5 (4.5–6.4)	0.613
Lymphocytes × 10^9^/L (normal range: 1.0–3.0)	1.7 (1.2–2.3)	1.7 (1.2–2.2)	1.6 (0.9–2.5)	1.9 (1.9–2.0)	0.643
NLR (normal range: 1–3)	3.1 (1.9–4.5)	3.2 (1.9–4.5)	2.7 (1.9–4.9)	2.8 (2.3–3.4)	0.815
Hemoglobin, g/dL (normal range: 12.0–15.0)	13.4 (12.2–14.5)	13.6 (12.3–14.5)	12.6 (12.2–14.8)	12.5 (11.6–13.3)	0.574
Platelets × 10^9^/l (normal range: 150–410)	203 (172–241)	213 (173–250)	195 (174–206)	155 (133–177)	0.164
TSH, uU/mL (normal range: 0.35–4.94)	1.7 (0.8–2.9)	1.7 (0.8–2.9)	1.5 (0.8–3.9)	1.8 (1.8–1.9)	0.961
FT4, pmol/L (normal range: 9.01-19.05)	15.3 (13.4–16.4)	15.2 (13.6–16.3)	14.4 (12.0–16.7)	17.4 (17.4–17.4)	0.495
ALT, UL (normal range: 5–34)	25 (19–37)	27 (22–40)	15 (14–24)	187 (44–330)	**0.005**
AST, U/L (normal range: <55)	24 (18–29)	25 (18–30)	22 (17–23)	39 (20–58)	0.346

ALT: alanine aminotransferase, AST: aspartate aminotransferase, BNP: brain natriuretic peptide, CK-MB: creatine kinase-myocardial band, CRP: C-reactive protein, FT4: free thyroxin, hsTnI: high‐sensitivity troponin I, NLR: neutrophil to lymphocyte ratio, TSH: thyroid stimulating hormone, nd: no data. Bold values are statistically significant values.

**Table 4:** Laboratory parameters before radiofrequency ablation (RFA) for ventricular electrical storm depending on the long-term ablation result.

	Study population (*n* = 62)	Long-term effective ablation (*n* = 19)	Long-term partially successful ablation (*n* = 15)	Long-term ineffective ablation (*n* = 13)	*p* value
hsTnI, ng/mL (normal range: <0.0156 for women; <0.0342 for man)	0.053 (0.029–0.150)	0.037 (0.023–0.117)	0.048 (0.030–0.096)	0.079 (0.036–0.199)	0.308
CK-MB, ng/mL (normal range: <3.1)	1.4 (0.9–2.6)	1.0 (0.7–1.3)	2.6 (1.5–4.0)	1.4 (1.2–1.5)	**0.027**
BNP before RFA, pg/mL (normal range: <159)	261 (145–728)	146 (67–220)	330 (156–365)	1029 (255–1677)	**0.004**
BNP at hospital discharge, pg/mL (normal range: <159)	354 (176–495)	135 (129–156)	302 (190–393)	369 (242–408)	**0.029**
Creatinine, mg/dL (normal range: 0.55–1.02)	1.17 (1.0–1.47)	1.06 (0.94–1.19)	1.22 (1.0–1.37)	1.26 (1.12–1.29)	0.417
Potassium, mmol/L (normal range: 3.5–5.1)	4.3 (4.0–4.5)	4.4 (4.1–4.8)	4.2 (4.0–4.4)	4.2 (4.0–4.6)	0.209
CRP, mg/L (normal range: <5.0)	3.4 (1.2–13.3)	2.6 (1.2–7.8)	4.7 (1.6–32.3)	1.5 (0.6–14.9)	0.419
Neutrophils × 10^9^/L (normal range: 2.0–7.0)	4.9 (3.6–6.6)	4.7 (3.5–6.5)	4.7 (3.4–5.5)	5.3 (4.5–6.4)	0.528
Lymphocytes × 10^9^/L (normal range: 1.0–3.0)	1.7 (1.2–2.3)	1.8 (1.5–2.6)	1.5 (1.1–1.8)	1.7 (0.7–1.9)	0.064
NLR (normal range: 1–3)	3.1 (1.9–4.5)	2.3 (1.8–4.0)	3.2 (2.2–4.8)	3.7 (2.3–6.5)	0.145
Hemoglobin, g/dL (normal range: 12.0–15.0)	13.4 (12.2–14.5)	13.9 (12.6–14.6)	13.6 (12.3–14.5)	12.5 (12.0–14.2)	0.490
Platelets × 10^9^/l (normal range: 150–410)	203 (172–241)	212 (189–250)	219 (197–338)	177 (170–187)	**0.014**
TSH, uU/mL (normal range: 0.35–4.94)	1.7 (0.8–2.9)	2.2 (0.9–3.5)	2.4 (0.9–3.3)	1.8 (0.7–2.0)	0.545
FT4, pmol/L (normal range: 9.01-19.05)	15.3 (13.4–16.4)	14.6 (13.6–15.4)	15.3 (15.2–16.3)	15.5 (14.6–18.1)	0.428
ALT, UL (normal range: 5–34)	25 (19–37)	23 (19–26)	41 (25–51)	26 (14–59)	0.116
AST, U/L (normal range: <55)	24 (18–29)	21 (16–24)	33 (28–46)	26 (21–33)	**0.003**

ALT: alanine aminotransferase, AST: aspartate aminotransferase, BNP: brain natriuretic peptide, CK-MB: creatine kinase-myocardial band, CRP: C-reactive protein, FT4: free thyroxin, hsTnI: high‐sensitivity troponin I, NLR: neutrophil to lymphocyte ratio, TSH: thyroid stimulating hormone.

**Table 5:** Predictors of the long-term ineffective radiofrequency ablation (RFA) for ventricular electrical storm evaluated by the Cox proportional hazards regression model.

Variables	Univariate analysis			Multivariate analysis		
	*p* value	HR	95% CI	*p* value	HR	95% CI
Age, years	0.101	1.07	0.99–1.17	0.66	1.03	0.9–1.17
ICM	0.125	0.36	0.1–1.33	**0.009**	0.05	0.01–0.48
LVEF, %	0.08	0.92	0.83–1.01	0.433	0.95	0.83–1.08
GFR < 60 mL/min/1.73 m^2^	0.075	3.35	0.89-12.63	**0.015**	16.94	1.74–165.04
NLR	0.088	1.19	0.98–1.45	**0.017**	1.35	1.05–1.72
BNP, pg/mL	0.252	1.0	1.0–1.01			
hsTnI, ng/mL	0.649	0.65	0.1–4.18			
Short-term complete RFA success	0.03	0.2	0.05–0.85	**0.011**	0.03	0.01–0.44

95% CI: 95% confidence interval, BNP: brain natriuretic peptide, GFR: glomerular filtration rate, HR: hazards ratio, hsTnI: high‐sensitivity troponin I, ICM: ischemic cardiomyopathy, LVEF: left ventricular ejection fraction, NLR: neutrophil to lymphocyte ratio. Bold values are statistically significant values.

## Data Availability

The data used to support the findings of this study are available on request from the corresponding author.

## Supplementary Materials

Supplementary Materials Supplementary Figure 1: computed tomography 3D reconstruction and scan showing a patient undergoing RF ablation with dextrocardia and visceral inversion. Central illustration: risk factors of effective and ineffective ventricular electrical storm ablation.
