# Supplementary material for: Clinical and Laboratory Predictors of Long-Term Outcomes after Catheter Ablation for a Ventricular Electrical Storm
Source: J Interv Cardiol. 2024 Feb 5;2024:5524668. doi: 10.1155/2024/5524668 (PMC10861284; doi:10.1155/2024/5524668)

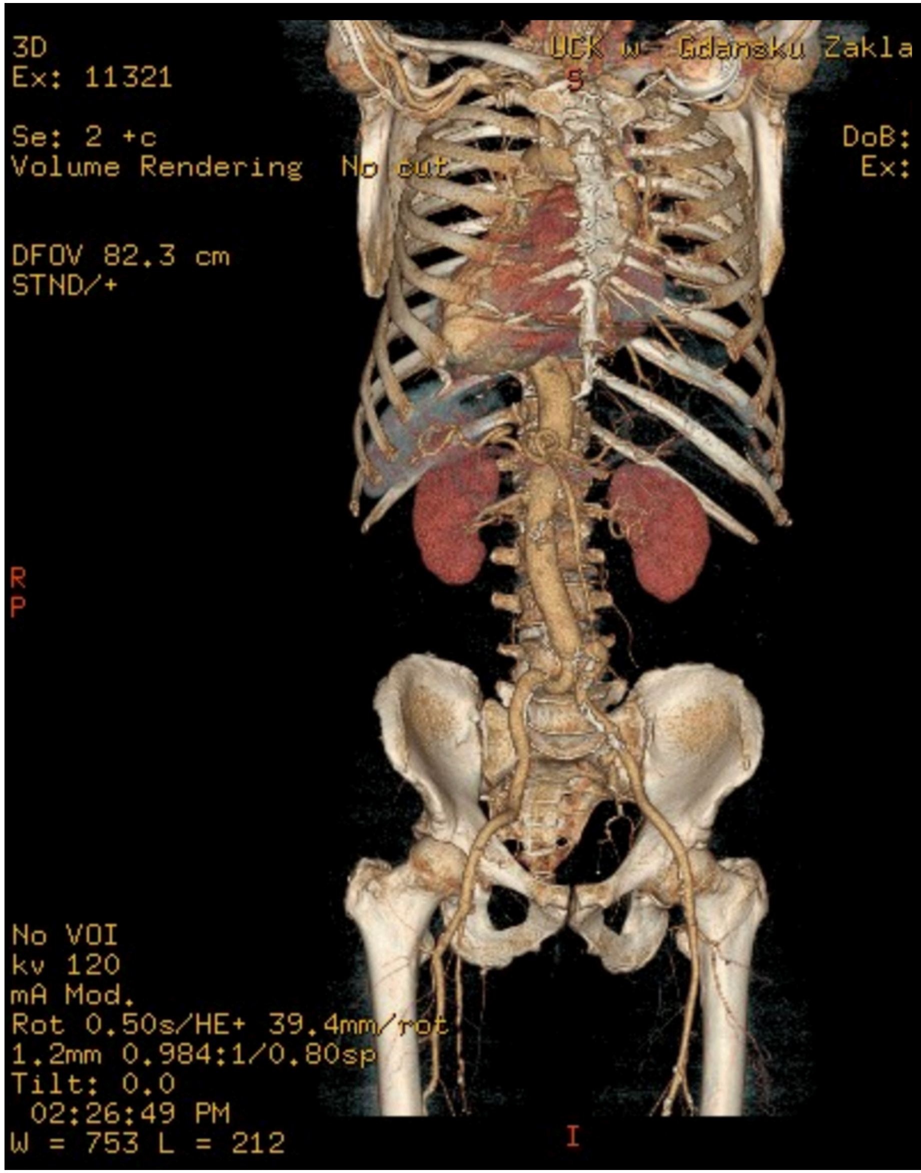
3D
Ex: 11321
UCK w Gdańsku Zakla
S
Se: 2 +c
Volume Rendering No cut
DoB:
Ex:
DFOV 82.3 cm
STND/+
R
P
No VOI
kv 120
mA Mod.
Rot 0.50s/HE+ 39.4mm/rot
1.2mm 0.984:1/0.80sp
Tilt: 0.0
02:26:49 PM
W = 753 L = 212
I

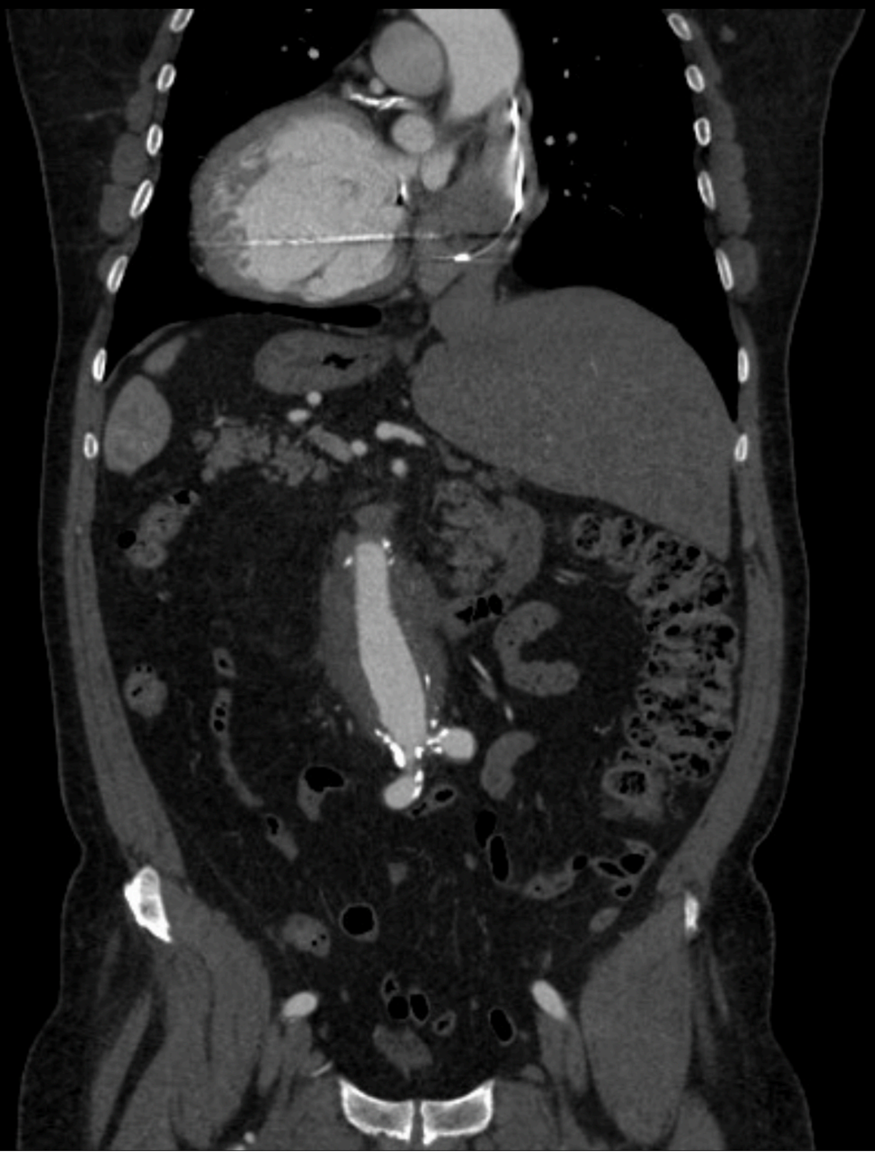

Supplement: Supplementary Materials — Supplementary Figure 1: computed tomography 3D reconstruction and scan showing a patient undergoing RF ablation with dextrocardia and visceral inversion. Central illustration: risk factors of effective and ineffective ventricular electrical storm ablation. [file 5524668.f1.zip › Supplementary Figure 1.pdf]
