# Supplementary figures and images for: Clinical and Laboratory Predictors of Long-Term Outcomes after Catheter Ablation for a Ventricular Electrical Storm
Source: J Interv Cardiol. 2024 Feb 5;2024:5524668. doi: 10.1155/2024/5524668 (PMC10861284; doi:10.1155/2024/5524668)

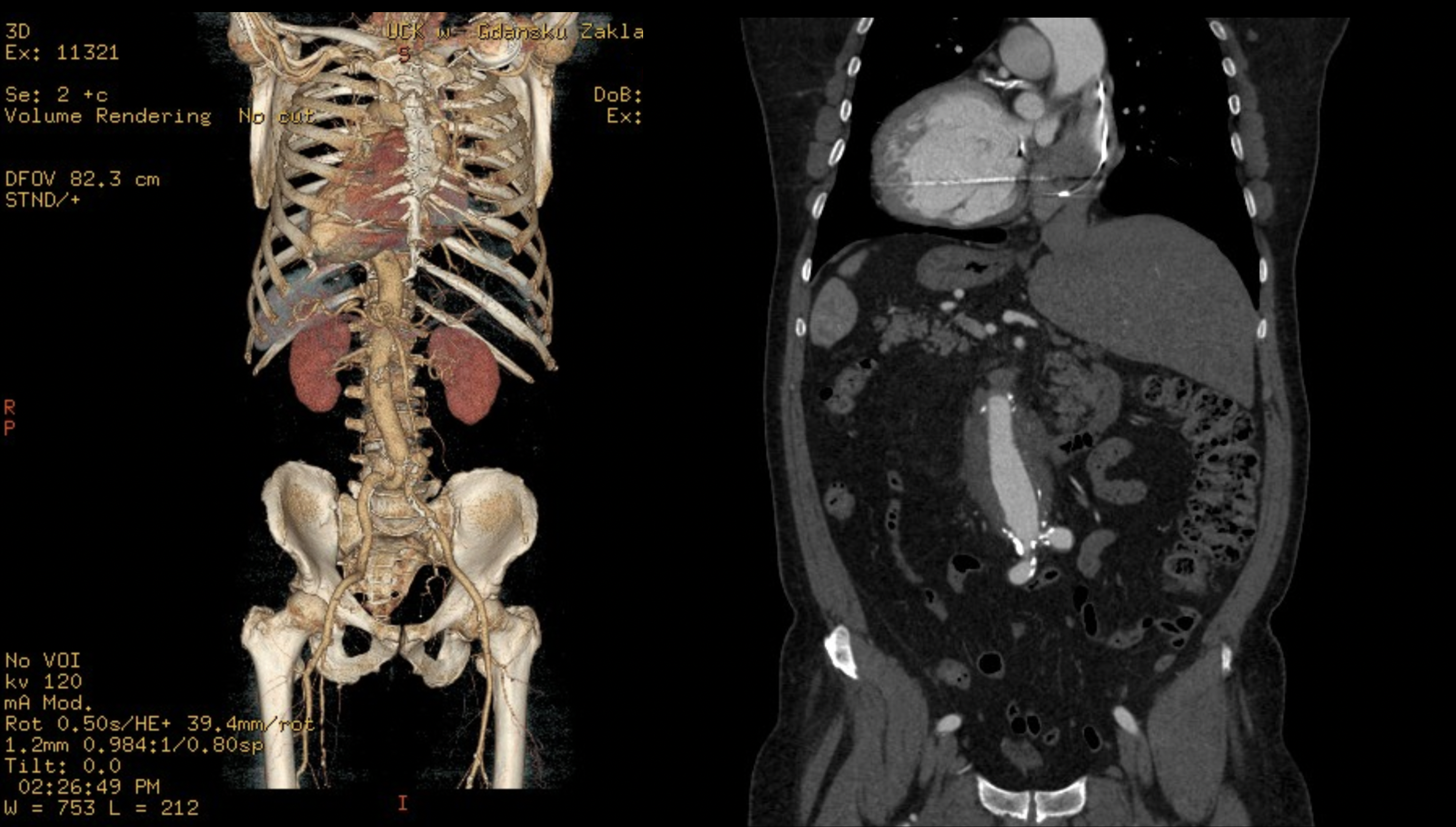

Supplement: Supplementary Materials — Supplementary Figure 1: computed tomography 3D reconstruction and scan showing a patient undergoing RF ablation with dextrocardia and visceral inversion. Central illustration: risk factors of effective and ineffective ventricular electrical storm ablation. [file 5524668.f1.zip › Supplementary Figure 1.png]
